# Diagnostic value of ultrasonographic features in breast cancer and its correlation with hormone receptor expression

**DOI:** 10.3389/fonc.2025.1538775

**Published:** 2025-08-13

**Authors:** Jingzhou Yang, Xiaojuan Yu, Jianxin Liu

**Affiliations:** Department of Ultrasound, The Central Hospital of Wuhan, Tongji Medical College, Huazhong University of Science and Technology, Wuhan, Hubei, China

**Keywords:** ultrasound elastography, breast cancer, diagnostic value, hormone receptor expression, malignant breast lesions

## Abstract

**Introduction:**

The objective of this research is to investigate the diagnostic value of Ultrasonographic characteristics in breast cancer (BC) and its relation to hormone receptor status.

**Method:**

Patients who underwent Breast Neoplasm surgery from January 2020 to December 2022 were included, 68 patients with malignant and 40 patients with benign, based on pathological diagnosis as the reference standard. Each patient volunteered for an elastography imaging examination, and one lesion was evaluated for each patient.

**Results:**

Patients had with mean age of 53.57 years (± 6.34), and lesion diameters ranged from 0.53-2.5 cm (1.29 cm ± 0.41). Kappa consistency analysis examined the inter-observer reliability of ultrasound elasticity signs in discriminating malignant from benign neoplasms. The correlation between ultrasonographic features and hormone receptor activity was also determined using Spearman correlation. Possible factors for having malignant BC lesions were tested, and binary logistic regression was utilized to develop the nomogram prediction model. There the malignant and benign groups were statistically significant in terms of ER, PR, Ki-67, HER-2 expression, marginal burrs, calcification foci, lesion diameter, and blood flow signal grading (P-levels < 0.05). The results of elastography indicated substantial concordance with pathological diagnosis (kappa = 0.875; P < 0.01). Statistically significant, positive correlations between breast cancer lesion diameter and Ki-67, burr sign and ER, as well as blood flow signal grading with PR, ER, Ki-67 and HER-2 were also observed. The independent predictors of malignancy in multivariate analysis included calcifications burr sign blood flow grading elasticity score ER and HER-2 (P < 0.05). By analyzing comprehensive clinical characteristics and measuring the total score as 263 points, the nomogram model achieved high validity in predicting the malignancy of breast lesions and could be applied in daily work with a 74.12% predicted probability of malignancy.

**Discussion:**

The performance of ultrasound elastography is promising for BC diagnosis, and the association with hormone receptor status is highly significant. Further confirming that the elasticity score has significant clinical value for the diagnosis of malignant breast lesions, in addition to the fact that hormone receptor levels are independent of elasticity score, we can conclude that the elasticity score and hormone receptor levels are two distinct factors for malignant lesions.

## 1 Introduction

Breast disorders consist of inflammatory conditions, benign irregularities, and malignant cancers. The prevalence and death rates of breast cancer in China are expected to rise for the foreseeable future. Population-based screening enables the early proof of identity, evaluation, and treatment of BC, leading to a 5-year survival rate of 90% among patients diagnosed at an early stage (1). Ultrasound serves as a standard diagnostic tool for BC patients, providing advantages such as the lack of radiation exposure, non-invasive nature, cost-effectiveness, and the capability for real-time dynamic observation. Ultrasound elastography, as an emerging technology, can accurately assess the stiffness of breast tissue. Ultrasound elastography demonstrates higher efficacy in diagnosing intraductal BC and differentiating breast lesions from benign masses compared to conventional ultrasound in comprehensive diagnosis (2). Pathologic examination has been the definitive standard for cancer diagnosis, encompassing the clarification of etiology, pathogenesis, clinicopathologic correlation, and prognosis prediction (3). Clinical data indicate that molecular biology-based typing is essential for selecting the most suitable treatment and is highly beneficial for individualized therapy. Hormone receptor expression is crucial for individual treatment decisions (4), and endocrine and targeted therapies have been aimed at malignancies exhibiting favorable expression of these genetic markers. Early detection and differentiation diagnosis of highly malignant BC are essential for informing prognosis and guiding medical care for patients. This study evaluated the medical significance of ultrasonographic traits for BC and their association with hormone receptor expression.

## 2 Materials and methods

### 2.1 General information

A retrospective analysis was performed on data from 108 BC patients admitted to our hospital from February 2020 to May 2022. Patients were categorized into malignant (n = 68) and benign (n = 40) groups according to pathological diagnosis, which served as the gold standard. All patients underwent elastography and presented with a single lesion. The mean age of the patients was 53.57 ± 6.34 years, and the lesion diameter ranged from 0.53 to 2.5 cm, with a mean of 1.29 ± 0.41 cm. Ethical approval was obtained for the study, and informed consent was secured from all participants.

### 2.2 Inclusion and exclusion criteria

Inclusion criteria: (1) patients with pathologically verified benign or malignant tumors; (2) patients undergoing ultrasound elastography; (3) patients with a left ventricular ejection fraction of ≥ 50% and sufficient bone marrow, renal, and hepatic function; (4) patients who were informed about the study, whose participation was not opposed by themselves or their families, and who had previously signed the necessary protocol. Exclusion criteria: (1) Pregnancy, menstrual cycle, mild to extreme anemia, or mysterious vaginal hemorrhage; (2) those who have additional malignant neoplasms; and (3) those with serious conditions of critical organs (e.g., heart, liver, and kidneys) or those with insufficient clinical data.

### 2.3 Ultrasound methods

An initial routine ultrasound was conducted with an HV800 ultrasound machine before the tumor biopsy to assess tumor morphology, borders, aspect ratio, adjacent tissues, and echogenicity. This was succeeded by color Doppler flow imaging utilizing an HI VISION Ascendus ultrasound system (GE730, GE, USA) to evaluate tumor morphology, distribution, spectral patterns, and blood flow signals. Following that, ultrasound elastography was conducted, revealing that the specified area of interest was more than twice the size of the tumor. In this study, strain elastography was employed to evaluate the stiffness characteristics of breast lesions. This modality estimates tissue deformation in response to externally applied compression, allowing qualitative assessment through a five-point scoring system. The tumor region experienced limited vibration at a 3-4 Hz frequency, with the probe entering the subcutaneous layer of skin adjacent to the muscle layer. The probe is oriented perpendicular to the patient’s intact skin, with immediate modifications made to ensure vertical alignment.

**Figure 1:**
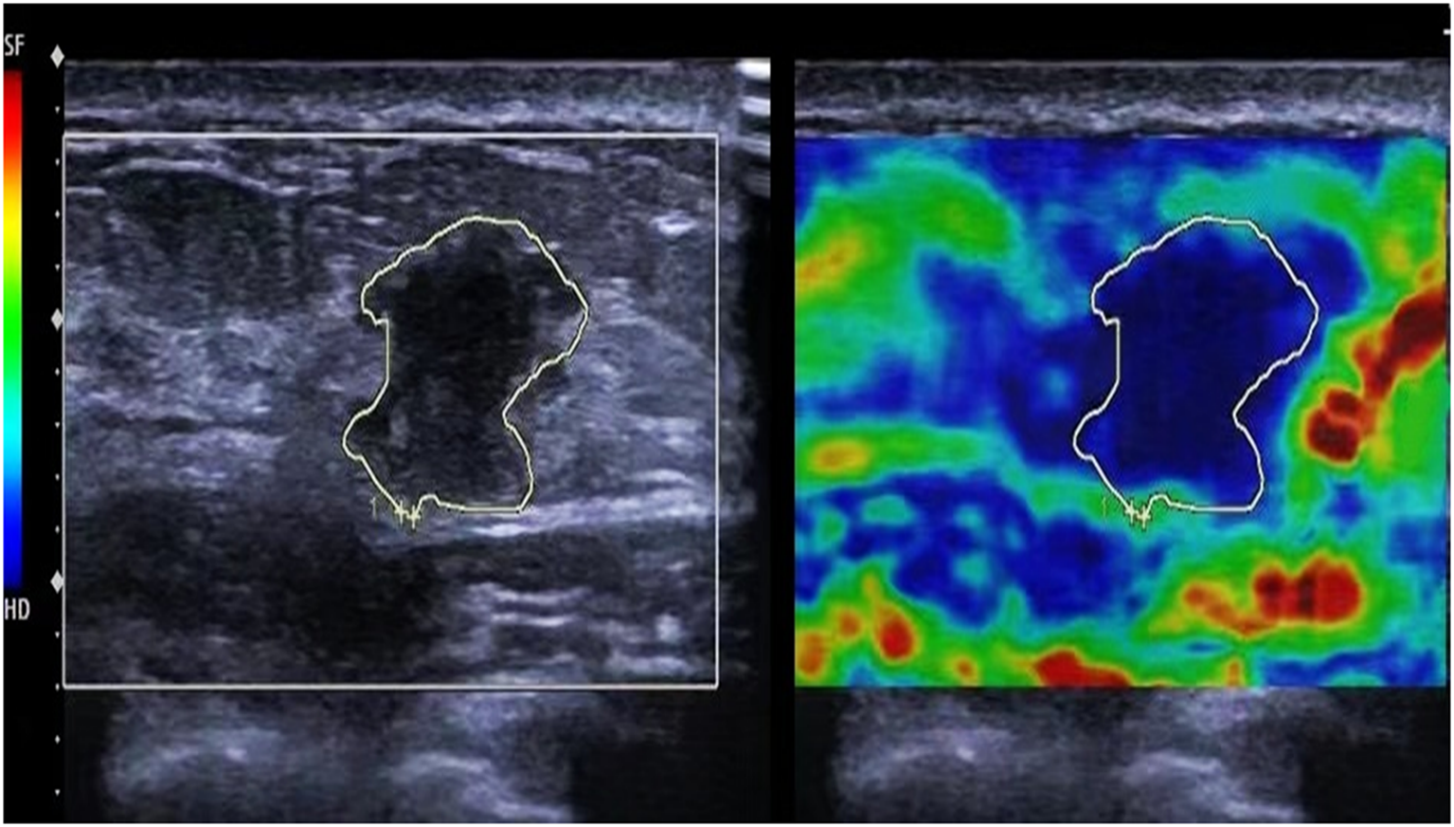
Ultrasound elastography of the patient. The tumor and surrounding tissues appearing blue with no deformation, exhibiting an ultrasound elastography elasticity score of 5.

The images were evaluated using a five-point Tsukuba elasticity scoring system, as illustrated in **Figure 1**. 1 point was assigned to an entirely green image, indicating a fully deformed tumor; 2 points for a predominantly green image with visible blue, suggesting that most of the tumor was deformed while a small portion remained intact; 3 points for an image where the tumor’s exterior was green and deformed, but the central area was blue and intact; 4 points for an overall blue image of the tumor with no deformation; and 5 points for a combination of the tumor and surrounding tissues appearing blue with no deformation. Scores of 1-3 indicate modest elasticity, whereas scores of 4-5 signify high elasticity, suggesting the existence of malignant tumors. In addition to the elasticity score, all ultrasound images were evaluated and classified using the Breast Imaging Reporting and Data System (BI-RADS). The BI-RADS category was determined based on lesion shape, margin, orientation, echogenicity, and posterior features, as observed on conventional ultrasound. This classification was used to complement the elastography findings and provide a standardized risk stratification for breast lesions.

### 2.4 Observational indicators and detection methods

Clinical data of the patients were obtained, containing information such as age, menstruation status, co-morbidities, and concurrent disorders. All patients underwent core needle biopsy before treatment, with specimens collected for analysis. Immunohistochemistry was conducted to assess the expression of progesterone receptor (PR), estrogen receptor (ER), oncogene P53, proto-oncogene Bcl-2, proliferating cell nuclear antigen (Ki-67), and human epidermal growth factor receptor (HER2) both before and after treatment. All ultrasound images were evaluated by a senior sonographer with over five years of experience, who appraised the location, echogenicity, and size of breast lesions ultrasonographically. Thereafter, the blood flow within and surrounding the lesion was assessed using color Doppler flow imaging.

### 2.5 Statistical analysis

Statistical analysis was conducted utilizing SPSS 22.0 software, with count data represented as percentages (%). Group differences were assessed using the χ2 test; measurements were presented as `x ± s. Within-group comparisons employed the paired-samples t-test, while between-group comparisons utilized the independent-samples t-test. The diagnostic procedures’ reliability was assessed by “gold standard” Kappa consistency analysis, with kappa values indicating low consistency (<0.40), medium consistency (0.40-0.75), and good consistency (>0.75). Spearman’s rank correlation analysis was employed to examine correlations between indicators, with a significance level of α=0.05 and P<0.05 indicating statistical significance. Multivariate logistic regression was employed to examine the risk factors influencing malignant lesions. A nomogram prediction model was developed and internally validated utilizing the Bootstrap function in R software. The model’s discriminatory ability was assessed through receiver operating characteristic (ROC) curves, while the cumulative gain plot was utilized to evaluate the model’s practical applicability.

## 3 Results

### 3.1 Comparison of the clinical data of the two groups of patients

The analysis demonstrated substantial differences between the malignant and benign groups regarding ER and PR status, Ki-67 and HER-2 expression levels, the presence or absence of marginal burrs, calcified foci, mass diameter, and blood flow signal grading (P < 0.05). These differences were independent of age, comorbidities, menstrual status, the number of concomitant diseases, and the expression levels of P53 and Bcl-2 (P > 0.05), as shown in **Table 1**. In addition, BI-RADS categories were assigned to each lesion during ultrasound evaluation. Higher BI-RADS categories were more frequently observed in the malignant group compared to the benign group, supporting the utility of this standardized classification in risk assessment.

**Table 1:** Comparison of the clinical data of the two groups of patients.

Indicators	Grouping	Benign group(n=40)	Malignant group(n=68)	*χ* ^2^	*P*
Age (years)	<50	18 (45.00)	31 (45.59)	0.004	0.953
	≥50	22 (55.00)	37 (54.41)		
Menstrual status	Premenopausal	21 (52.50)	33 (48.53)	0.159	0.690
	Postmenopausal	19 (47.50)	35 (51.47)		
Comorbidities	Diabetes	14 (35.00)	20 (29.41)	0.710	0.871
	Hypertension	11 (27.50)	22 (32.35)		
	Neurological disease	8 (20.00)	16 (23.53)		
	Circulatory system disease	7 (17.50)	10 (14.71)		
Number of concomitant diseases	≤3	23 (57.50)	38 (55.88)	1.805	0.179
	>3	17 (42.50)	30 (44.12)		
ER	Negative	19 (47.50)	16 (23.53)	6.606	0.010
	Positive	21 (52.50)	52 (76.47)		
PR	Negative	16 (40.00)	13 (19.12)	5.607	0.018
	Positive	24 (60.00)	55 (80.88)		
P53	Negative	22 (55.00)	26 (38.24)	2.867	0.090
	Positive	18 (45.00)	42 (61.76)		
Bcl-2	Negative	21 (52.50)	35 (51.47)	0.011	0.918
	Positive	19 (47.50)	33 (48.53)		
Ki-67	Low expression	23 (57.50)	22 (32.35)	6.553	0.010
	High expression	17 (42.50)	46 (67.65)		
HER-2	Negative	19 (47.50)	16 (23.53)	6.606	0.010
	Positive	21 (52.50)	52 (76.47)		
Edge burrs	Yes	9 (22.50)	36 (52.94)	9.602	0.002
	No	31 (77.50)	32 (47.06)		
Calcified foci	Yes	10 (25.00)	35 (51.47)	7.261	0.007
	No	30 (75.00)	33 (48.53)		
Mass diameter (cm)	<2.0	31 (77.50)	29 (42.65)	12.390	<0.001
	≥2.0	9 (22.50)	39 (57.35)		
Blood flow signal grading	0-I	34 (85.00)	39 (57.35)	8.788	0.003
	II-III	6 (15.00)	29 (42.65)		
Elastography score (points)	1-3	28 (70.00)	12 (17.65)	29.601	<0.001
	4-5	12 (30.00)	56 (82.35)		

### 3.2 Comparison of the consistency of diagnostic methods

Elastography demonstrated a high level of concurrence with the histological diagnosis, regarded as the “gold standard” (kappa value = 0.875, P < 0.01), indicating its reliability in distinguishing between benign and malignant breast lesions, as shown in **Table 2**.

**Table 2:** Comparison of the consistency of diagnostic methods.

Indicators	Pathology results		kappa	*P*
	Malignant	Benign		
Elastography results			0.875	<0.001
Malignant	56 (82.35)	12 (30.00)		
Benign	12 (17.65)	28 (70.00)		
Total	68	40		

### 3.3 Analysis of the correlation between ultrasonographic features and hormone receptor expression

Statistical analysis showed that the diameter of the mass had a positive correlation with Ki-67 expression (r=0.749, P<0.05), the burr sign had a positive correlation with ER expression (r=0.617, P<0.05), the blood flow signal grading had a positive correlation with PR, ER, Ki-67, and HER-2 expression, and the elasticity scores had a specific positive correlation with ER and HER-2 expression and data are shown in **Table 3**.

**Table 3:** Correlation analysis between ultrasonographic features and hormone receptor expression (r-value, P-value).

Indicators	PR	ER	Ki-67	HER-2
Mass diameter	(0.347, 0.083)	(0.125, 0.214)	(0.749, 0.000)	(0.302, 0.095)
Calcified foci	(0.138, 0.106)	(0.126, 0.212)	(0.241, 0.077)	(0.239, 0.074)
Edge burrs	(0.142, 0.101)	(0.617, 0.004)	(0.411, 0.057)	(0.235, 0.089)
Hemodynamic signal grading	(0.697, 0.000)	(0.742, 0.000)	(0.651, 0.002)	(0.733, 0.000)
Elastography score	(0.154, 0.167)	(0.369, 0.048)	(0.112, 0.161)	(0.515, 0.006)

### 3.4 Multivariate logistic analysis of malignant lesions

**Figure 2:**
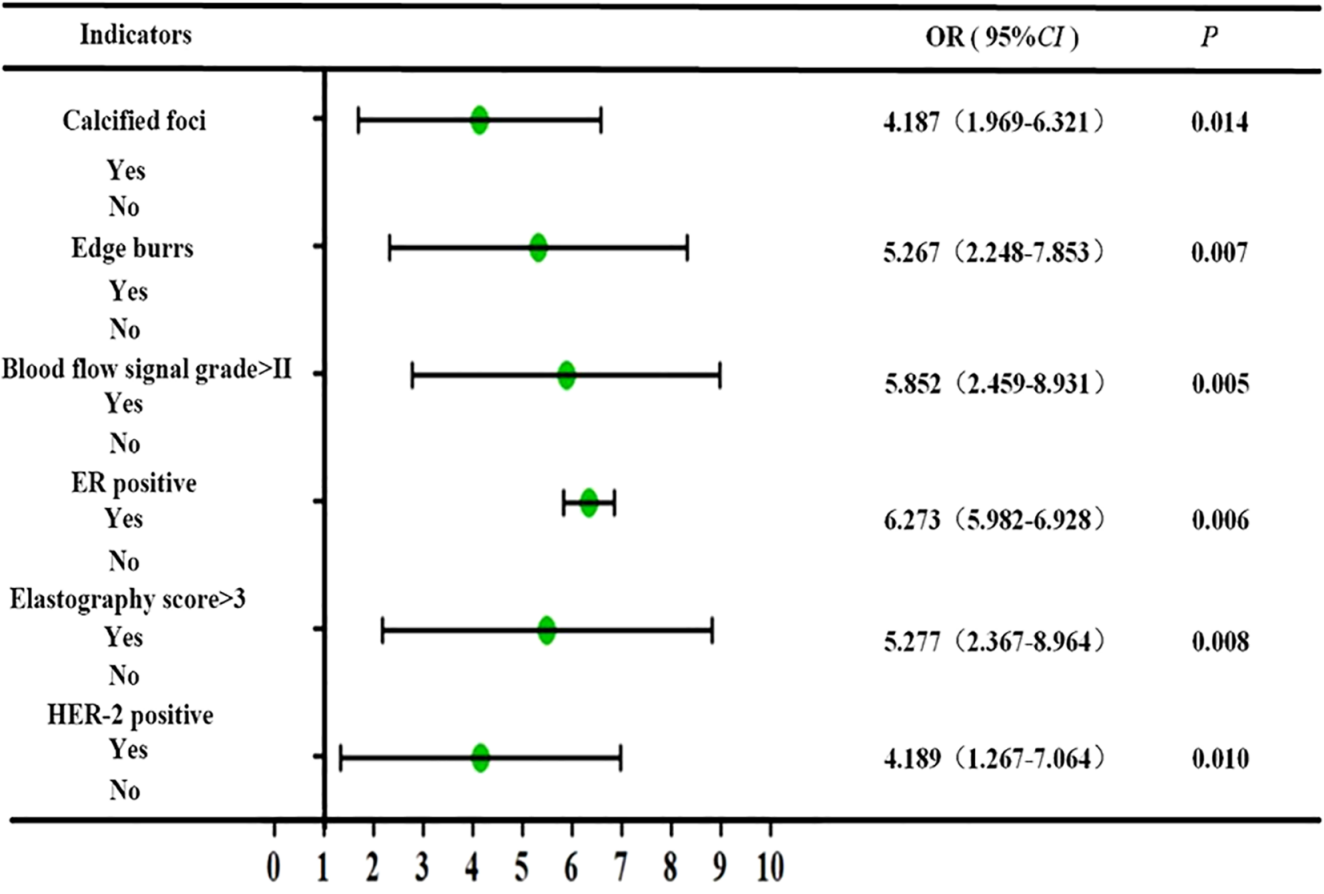
Forest map of risk factors for malignant lesions.

The results of multivariate analysis showed that calcified foci, burr sign, blood flow signal grading, elasticity score, and ER and HER-2 expression levels were independent risk factors for malignant lesions (P < 0.05), as shown in **Figure 2**.

### 3.5 Construction of nomogram prediction model

**Figure 3:**
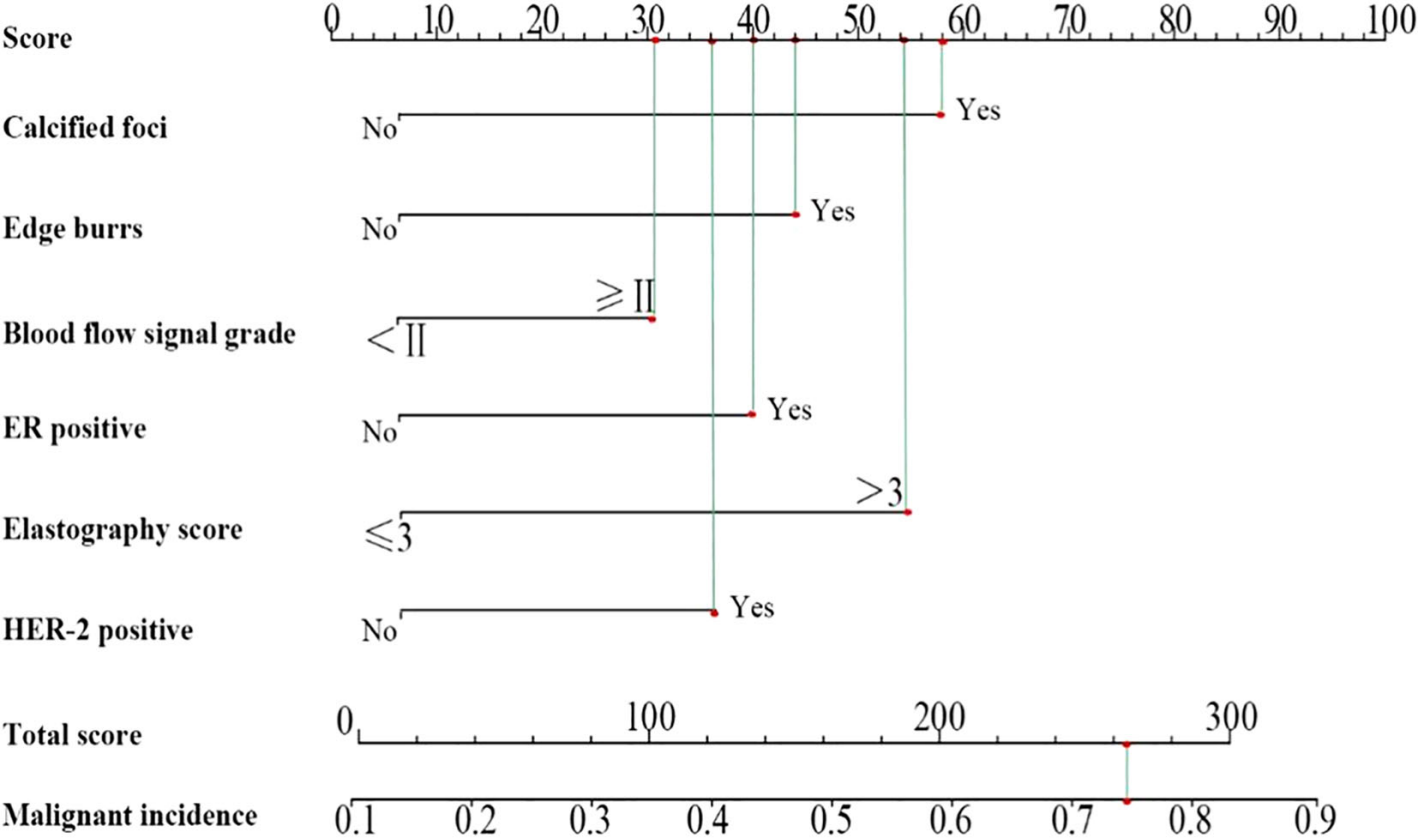
Nomogram prediction model.

According to the analysis results, a nomogram prediction model was developed, as shown in **Figure 3**. The scoring included 58 points for calcified foci, 44 points for burr sign, 31 points for blood flow signal grading, 40 points for ER positivity, 54 points for elasticity scoring, and 36 points for HER-2 positivity, culminating in a total score of 263 points, which corresponds to a 74.12% probability of malignant lesions.

### 3.6 Evaluation of the model

#### 3.6.1 Discriminatory degree of the model

**Figure 4:**
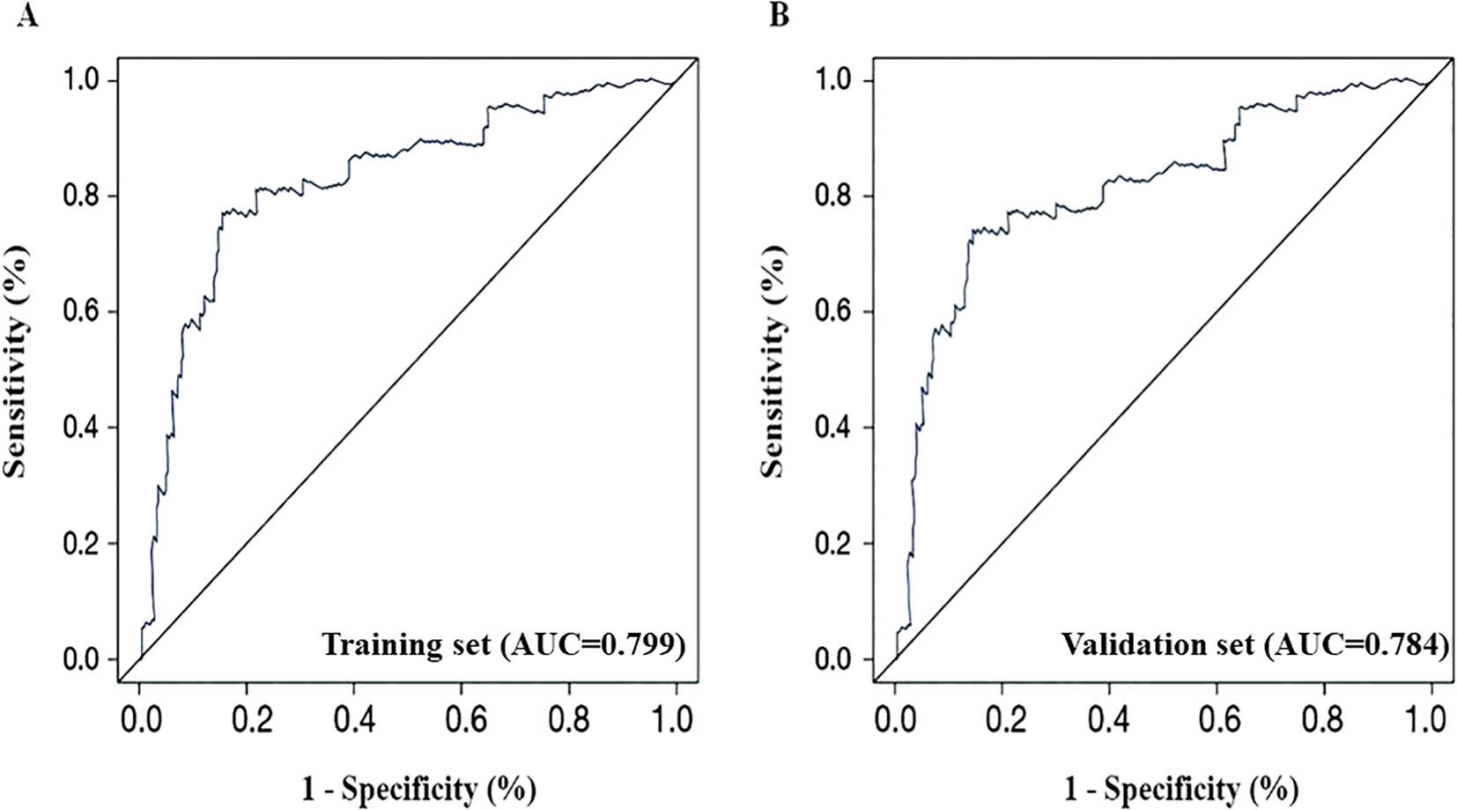
ROC curves of the predictive model. **(A)** training set; **(B)** validation set.

Internal validation of the patients demonstrated that the C-index of the training set was 0.815, while the C-index of the validation set was 0.803. The ROC curve was drawn to evaluate the discriminatory ability of the nomogram model. In the training set, as presented in **Figure 4A**, the AUC was 0.799, with a 95% CI ranging from 0.748 to 0.869 (P < 0.001). On the contrary, **Figure 4B** gives the AUC for the validation set as 0.784, 95% CI: 0.739-0.862, P < 0.001. To further characterize model performance, the Positive Predictive Value (PPV) was found to be 82.35%, and the F1-score was 0.83, indicating strong precision and balance between sensitivity and specificity in identifying malignant lesions. This also guarantees a great degree of model discrimination.

#### 3.6.2 Calibration evaluation

**Figure 5:**
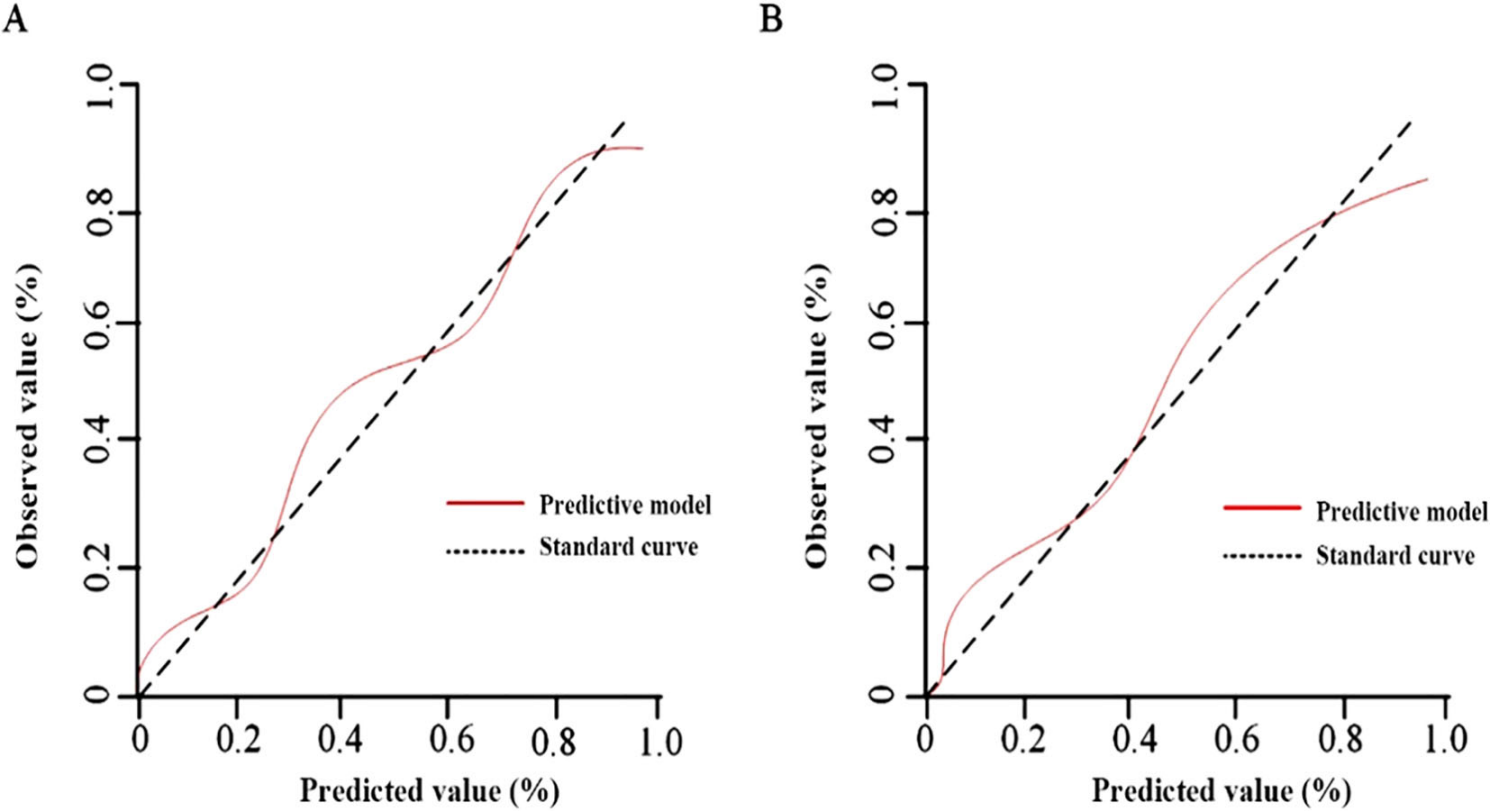
Calibration evaluation of the prediction model. **(A)** Training set; **(B)** Validation set.

The results of the Hosmer-Lemeshow goodness-of-fit test for the calibration curves showed no significant difference (P>0.05) in the training set χ2 = 1.036, P=0.242 (**Figure 5A**) and in the validation set χ2 = 2.025, P=0.109 (**Figure 5B**).

#### 3.6.3 Clinical decision curve analysis

**Figure 6:**
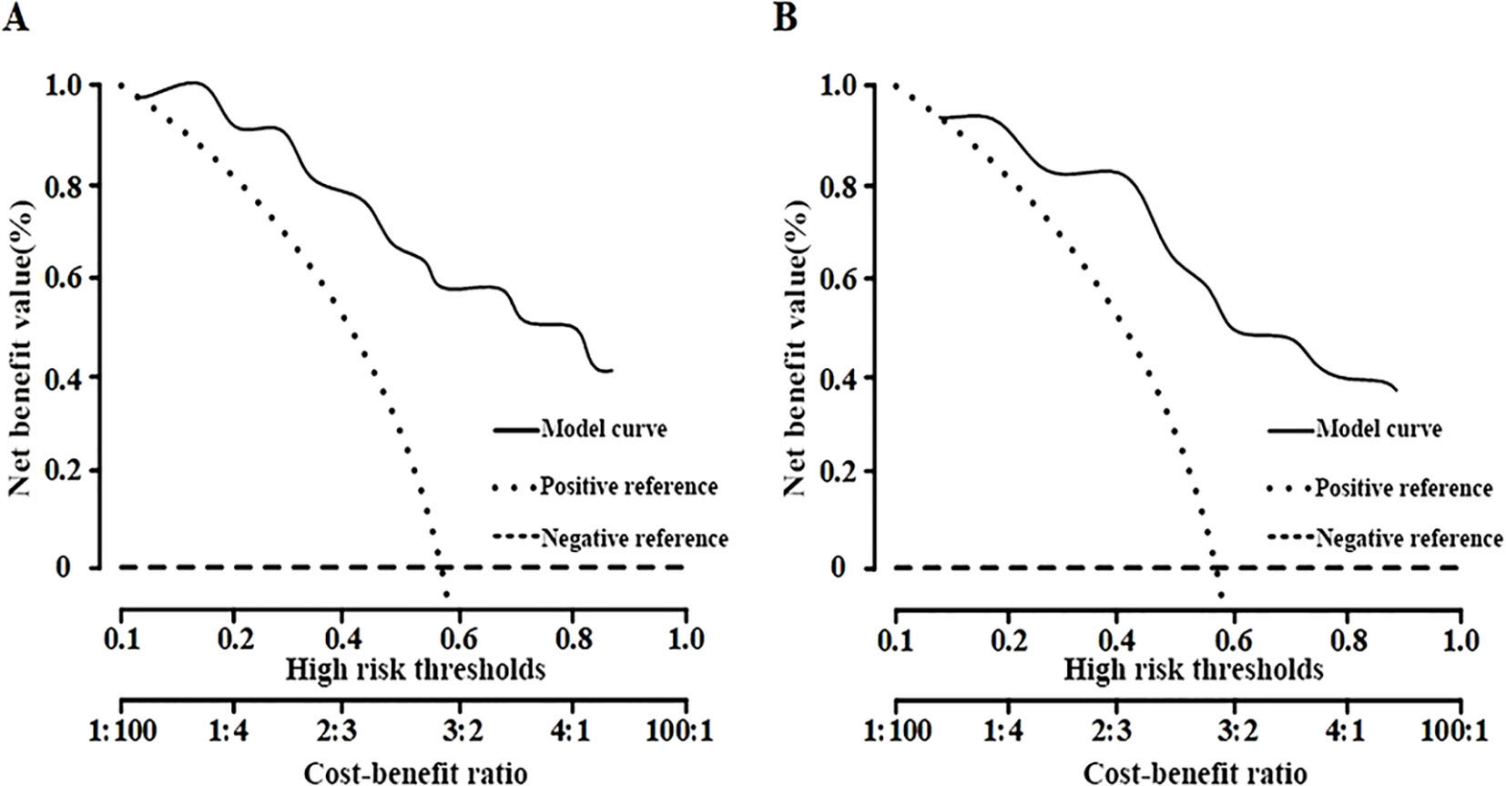
Decision curve analysis of the prediction model. **(A)** Training set; **(B)** Validation set.

The clinical decision curve analysis indicated that the net benefit of employing the nomogram for predicting malignant lesions was substantial in the training set, with threshold probabilities between 13% and 81% (**Figure 6A**). In the validation set, the net benefit remained significant within the threshold probability range of 16% to 85% (**Figure 6B**), thereby demonstrating the safety and practicality of the column-line diagram model.

To improve the clinical utility of the nomogram, we propose a practical threshold probability of ≥70% as a guideline for recommending biopsy. Lesions with predicted probabilities below this threshold may be considered for short-term imaging follow-up rather than immediate invasive procedures, thereby aiding in risk-adapted clinical decision-making.

## 4 Discussion

The progression of BC comprises the aberrant activation of oncogenes and the silencing of tumor suppressor genes. The subtle development of the condition and the absence of typical early symptoms in BC sometimes result in it being underestimated, leading to frequent misdiagnoses until apparent clinical manifestations emerge, which causes a delayed diagnosis (5). But cancer or metastasis often arises in some people, presenting a difficulty for therapy. Early diagnosis and precise evaluation of prognosis may substantially enhance the quality of life for patients. Molecular diagnostics has emerged as a novel approach for the diagnosis of BC (6). Different genetic testing criteria possess distinct interpretations and varying relationships with the clinical or pathological characteristics of BC. Ultrasound elastography, an emerging technology, has developed into a functional imaging modality relative to conventional ultrasound. By utilizing tissue elasticity coefficients and digital signal processing techniques, it enhances the clinical evaluation of tissue stiffness. Ultrasound elastography provides a distinct advantage in differentiating between benign and malignant tumors (7).

It thus holds great value for the classification of BC and subsequently for a decision on the regimen of therapy. So far, many molecular markers have already been put into clinical use in the diagnosis of BC, and the main tumor markers comprise glycoantigen 153, glycoantigen 125, carcinoembryonic antigen, and HER2, the expression of which is crucial in the treatment of BC (8, 9). Therefore, in the process of identification and diagnosis of BC, the expression of hormone receptors may act as a prognostic factor for patients. It is mostly based on a biopsy specimen or surgically obtained immune tissues. However, during clinical assessment, biopsy or surgical sample for hormone receptor expression has certain limitation. It can neglect the heterogeneity of some cancers (10). Relevant research indicates that imaging facilitates a more thorough analysis of tumor tissue, including the evaluation of internal echogenicity, form, size, and margin morphology of the tumor (11, 12). During the past decade, most of the studies reported on breast imaging have focused on the prediction of malignancy lesions or detection of additional lesions in patients scheduled to undergo surgery, with few studies reviewed regarding the agreement between the findings of imaging versus tumor characteristics. Irshad et al. (13) mentioned that posterior shadowing was significantly associated with ER-positive and low-grade cancer while posterior enhancement was significantly associated with the likelihood of a high-grade tumor and receptor negativity. Likewise, ultrasonographic enhancement kinetics were connected with the hormonal state of infiltrating BC, and increased stiffness on shear wave elastography was linked to unfavorable prognostic indicators (14). The nomogram developed in this study serves as a clinically interpretable decision-support tool, which can guide biopsy recommendations based on individualized malignancy risk. By establishing a ≥70% probability threshold, the model provides a rational basis for distinguishing cases that warrant immediate histopathological confirmation from those suitable for monitoring, ultimately aiming to reduce unnecessary biopsies and enhance personalized care.

The HER2 gene encodes a protein that preferentially binds to a ligand to create a heterodimer, resulting in a conformational alteration of the HER2 protein and the activation of signaling pathways via autophosphorylation of cytoplasmic tyrosine kinases. The activity of this heterodimer exceeds that of heterodimers generated by other members of the EGFR family, hence initiating a cascade reaction (15). HER2 proteins and ligands participate in cellular physiological functions, facilitating cell activation, proliferation, and differentiation. Certain studies indicate that HER2 expression correlates positively with clinical TNM stage, tumor size, and lymph node metastasis, while exhibiting a negative correlation with ER/PR expression and tissue grading (16). This suggests that HER2 expression could serve as a molecular marker for the diagnosis and prognostic evaluation of BC, thereby verifying the findings of the current study. Ki-67 is a prominent nuclear protein associated with tumor proliferation-related genes, indicating cell proliferation and strongly linked to BC differentiation and tumor metastasis. A study indicated a positive correlation between elevated Ki-67 expression and tumor diameter, lymph node metastasis (LNM), and blood flow grading (17), aligning with our results; another investigation revealed that Ki-67 expression was positively correlated with TNM staging and LNM, while negatively correlated with tissue grading and ER/PR expression (18).

This heterodimer activity is higher compared to the ones provided by other members of the EGFR family; thus, it triggers a cascade reaction accordingly. HER2 proteins and their ligands take part in the physiological activity of cells, including cell activation, proliferation, and differentiation (15). Some reports have suggested that HER2 expression was positively related to clinical TNM stage, tumor size, and lymph node metastasis while negatively related to ER/PR expression and tissue grading (16). These findings confirm that HER2 may be used as a molecular marker of diagnosis and prognosis of BC. Ki-67 is a kind of nucleus protein that is closely related to genes having proliferating tumors, reflecting cell proliferation and having a close relationship with the differentiation and metastasis of BC. Some studies have reported that elevated Ki-67 expression was positively correlated with tumor diameter, lymph node metastasis (LNM), and blood grading (17). Another study showed that Ki-67 expression was positively correlated with TNM staging and LNM, and negatively with tissue grading and ER/PR expression (19). One of the limitations of this study is the relatively modest AUC values (below 0.8), which may affect the strength of the model’s clinical applicability. This may be improved in future work by incorporating additional clinical variables such as genetic markers, mammographic findings, and family history to optimize the model’s discriminatory power. Another notable limitation of this study pertains to the standardization of elastographic techniques. Given that elastography technology varies across different equipment manufacturers and software platforms, discrepancies in strain scoring and elasticity measurements may arise. These technical variances could influence the model’s external validity and limit its immediate generalizability across institutions employing different ultrasonographic systems. Future multi-center studies incorporating diverse elastographic setups are warranted to validate and refine the nomogram’s robustness.

Conclusion: The study highlights that hormone-expressing receptors in BC are both autonomous and interconnected, and are pertinent to the ultrasonographic characteristics and diagnosis of the disease. The simultaneous detection of many indications enables a more precise assessment of BC progression, offering guidance for personalized targeted medication therapy. Nevertheless, owing to the constrained sample size and brief follow-up duration, it is essential to augment the sample size and prolong the follow-up period for a more comprehensive analysis, particularly for long-term prognosis.

## Data Availability

The raw data supporting the conclusions of this article will be made available by the authors, without undue reservation.
